# How visual information influences dual-task driving and tracking

**DOI:** 10.1007/s00221-020-05744-8

**Published:** 2020-02-08

**Authors:** Laura Broeker, Mathias Haeger, Otmar Bock, Bettina Kretschmann, Harald Ewolds, Stefan Künzell, Markus Raab

**Affiliations:** 1 Institute of Psychology, German Sport University Cologne, Am Sportpark Müngersdorf 6, 50933 Cologne, Germany; 2 Institute of Exercise Training and Sport Informatics, German Sport University Cologne, Am Sportpark Müngersdorf 6, 50933 Cologne, Germany; 3 Institute of Sports Science, Augsburg University, Universitätsstraße 3, 86135 Augsburg, Germany; 4 School of Applied Sciences, London South Bank University, 103 Borough Road, London, SE1 0AA UK

**Keywords:** Dual task, Predictability, Driving simulation, Manual tracking

## Abstract

The study examined the impact of visual predictability on dual-task performance in driving and tracking tasks. Participants (*N* = 27) performed a simulated driving task and a pursuit tracking task. In either task, visual predictability was manipulated by systematically varying the amount of advance visual information: in the driving task, participants drove at night with low beam, at night with high beam, or in daylight; in the tracking task, participants saw a white line that specified the future target trajectory for 200, 400 or 800 ms. Concurrently with driving or tracking, participants performed an auditory task. They had to discriminate between two sounds and press a pedal upon hearing the higher sound. Results show that in general, visual predictability benefited driving and tracking; however, dual-task driving performance was best with highest visual predictability (daylight), dual-task tracking performance was best with medium visual predictability (400 ms). Braking/reaction times were higher in dual tasks compared to single tasks, but were unaffected by visual predictability, showing that its beneficial effects did not transfer to the auditory task. In both tasks, manual accuracy decreased around the moment the foot pressed the pedal, indicating interference between tasks. We, therefore, conclude that despite a general beneficial impact of predictability, the integration of visual information seems to be rather task specific, and that interference between driving and audiomotor tasks, and tracking and audiomotor tasks, seems comparable.

## Introduction

### The impact of predictability in driving simulations

It is a matter of common knowledge that car driving requires the handling of multiple tasks at the same time, like lane and distance keeping while watching for other road users and cockpit gauges. On top of that, we perform additional tasks unrelated to the main driving activity: talking to the co-driver or illegally using the cell phone are just two examples. The negative consequences resulting from such multitasking are well documented (Strayer and Drews 2007). Costs occurring from distraction while driving can be longer reaction times in braking, impaired lateral steering control and, related to this, more crashes with other cars or objects (Caird et al. 2014; Consiglio et al. 2003; Drews et al. 2013; for classic dual-task costs see Pashler et al. 1998).

These costs may differ depending on various external conditions like traffic density or reduced visibility of the road (Mueller and Trick 2012; Trick et al. 2010), and external conditions themselves may have a different impact depending on their degree of predictability, e.g., higher density in traffic involving more unpredictable breaks of cars out in front, and darker environments involving less predictability in routing. This impact was tested by Lundqvist et al. (1997) who compared driving performance for high (good sight; straight roads; preceding car with slightly varying speed) and low predictability (sudden braking of preceding car, sudden appearance of parked car behind a curve; unexpected visual stimuli appearing in field of view) in patients with brain lesions and healthy controls. Both groups were interrupted three times by a distracting listening span task, mimicking phone calls while driving. In the predictable condition, there were no differences between groups for safety margins (time and space to preceding car), so patients drove as well as controls. In the unpredictable condition, patients showed longer reaction times, had an increased need for safety margins, and difficulties in allocating resources to a secondary task as indicated by fewer words recalled from the listening span task. However, the study did not test participants in single-task conditions, which makes it difficult to determine the extent of dual-task costs and also to evaluate the extent of the beneficial impact of predictability on dual-task requirements. In the same vein, Mueller and Trick (2012) demonstrated a beneficial impact of visual predictability in an expert–novice design, showing that novice drivers had higher hazard response times, greater steering variability, and were the only group to have collisions in a less predictable, foggy condition, but again single-task performance was not reported. As single-/dual-task differences are rarely considered in driving simulations (e.g. Konstantopoulos et al. 2010; Plainis and Murray 2002), it is hard to disentangle the differential impact of dual task (task-hindering) vs. predictability (task-promoting) conditions, and to explain where the benefits come from. It has been argued that prediction is an omnipresent principle of human behaviour and that the beneficial effects of predictability in the environment are universal (Blakemore et al. 2000; Broeker et al. 2017; Northoff 2014), yet evidence for this claim comes from mostly basic tasks.

### Beneficial impact of predictability from a classic dual-task perspective in basic motor tasks

On a theoretical basis, predictability in dual tasks decreases the overall need for resources because predictable tasks require fewer attentional resources or bind resources, and are processed more efficiently (Hazeltine et al. 2002; Król and Król 2017; Tombu and Jolicœur 2003; Trick et al. 2010; Wickens 2002). Because resource accounts suggest that multitasking costs occur due to competition for resources between concurrently performed tasks, resources made-available by predictability can be allocated to other tasks (Broeker et al. 2017). According to bottleneck theories (Pashler 2000), the response-selection bottleneck represents a filter that requires rapid channel switching when two tasks compete for response selection (or execution, see Bratzke et al. 2009). Predictability can improve central channel allocation because it enables preparatory processes which shorten the time for channel switching (De Jong 1995).

There is also empirical evidence for the impact of predictability in fairly basic continuous tasks. For instance, de Oliveira et al. (2014) showed that healthy participants’ performance on a steering task improved the longer visible path ahead of the cursor; in participants with developmental coordination disorder (DCD), steering performance was best described by a U-curved function with best performance in a 400 ms path condition. In the same vein, Broeker et al. (2020) showed that participants receiving similar advance visual information in a dual-task tracking paradigm, improved motor control despite the occurrence of distracting auditory sounds. It was noteworthy that when they provided participants with 200, 400, 600, 800 ms of information ahead, best performance (fewer tracking and response errors, plus faster response times) was achieved with 400 ms, so more information did not necessarily improve performance. Beyond that, they showed that manual control in tracking was disturbed (as indicated by increased tracking velocity) in the moment participants replied to sounds by pedal press, indicating some kind of motoric dual-task costs. These costs were, however, also lowest for 400 ms predictability.

To sum up, predictability has been shown to be beneficial in basic motor tasks and complex driving simulations, yet its contribution to interference reduction has only been demonstrated for simpler tasks and it is thus unclear whether predictability is a universally beneficial effect or rather task-specific operator. MacAdam (1981) and Strayer and Johnston (2001) stated that simulated driving tasks are comparable to tracking tasks and that driving can be labelled a pursuit tracking task, so principles of predictability in basic tasks should hold in more complex tasks, too. However, Wulf and Shea (2002) argued that principles derived from the learning of simple tasks cannot be transferred to the learning of complex tasks, so the debate affords further testing.

In this study, we, therefore, applied a within-participants design, examining the influence of predictability on both basic (tracking) and complex (driving) tasks under dual- and single-task conditions. In the interest of testing this impact of predictability, we tried to match two computerized tasks as much as possible. Participants performed a simulated driving task with three predictability conditions: driving by night with low beam, driving by night with high beam and driving in daylight. In dual-task conditions, they additionally had to discriminate between low-pitched distractor and high-pitched target sounds, and press a brake pedal upon hearing a target sound. In the basic motor task, participants performed a pursuit tracking task with 200, 400 or 800 ms predictability (as in Broeker et al. 2020) and for dual-task conditions performed the same auditory task as during driving. To compare predictability conditions in driving and tracking, we hypothesized low beam to be similar to 200 ms, high beam similar to 400, and daylight to 800 ms. Given that research with tracking tasks has shown that predictability was optimal for performance if it was 400 ms ahead, we would expect best dual-task performance in driving for high beam and 400 ms conditions. The underlying assumption is that “medium” visual predictability provides enough information for visuomotor control without the need for additional resources, because too much information requires increased visual processing and additional load that hampers dual-task performance (Marois and Ivanoff 2005).

## Method

### Participants

A total of 33 participants was recruited via participant databank, mailing list and advertisement sheets on a university campus (sample size estimation G-Power 3.1.9.2., repeated measures ANOVA, within factors, *f* = 0.25, *α* = 0.05, 1 − *β* = 0.90, *r* = 0.5, 30 participants). Pre-defined inclusion criteria required normal or corrected-to-normal vision, normal hearing ability, a minimum age of 18 years, and no self-reported musculoskeletal or neurological disorders. Two participants were excluded prior to data analysis due to incomplete data sets. After data analysis, another four participants were excluded as outliers because their driving performance (SDLP, see below) exceeded more than 3.29 SDs from the sample mean. This resulted in a total sample size of 27 participants (17 male and ten female; aged between 19 and 32 years, *M* = 24.52 years, SD = 2.97). Prior to the experiment, participants provided written informed consent and filled in a questionnaire asking for their dominant hand/foot, experience in using a joystick or driving simulator, driving licence, frequency of driving and multitasking behaviour during driving. After the experiment, participants were debriefed and compensated for participation (20 € or course credit and sweets). The study was approved by the local ethics committee and performed according to the 2008 Declaration of Helsinki.

### Task and display

The experiment consisted of a tracking and a driving paradigm. Half of the participants started with the driving paradigm and the other half performed the tracking paradigm first. Both paradigms were performed as single tasks (ST), which was only tracking/driving and only reacting to auditory signals, and as dual tasks (DT), which was tracking/driving together with reacting to auditory signals.

### Driving paradigm

#### Setup

Participants were seated in a commercially available driving simulator (Carnetsoft© BV, Groningen, NL), consisting of a computer (Windows 7, 64-bit system, Nvidia GTX 770 graphic card), three 48″ monitors (100 Hz each; total field of view = 195°), a car seat, a steering wheel, pedals on a sloped pedestal (clutch on the left, brake in the middle and gas on the right) and a gear shift. Steering wheel, seat, pedals and gear shift were positioned left from the centre of the middle screen to mimic real-world car driving. A black curtain surrounded the monitors and the test room was dimly lit to avoid potential distraction in the laboratory room. For reasons of comfort, the driver’s seat and pedals were adapted individually. Participants wore headphones (Sennheiser HD 65TV). The experimenter sat in a separated area of the test room to supervise compliance with the task.

#### Driving paradigm

Participants were instructed to drive on a given route at a constant speed of 100 km/h and to only use the right foot for controlling gas and brake pedals (automatic transmission). Participants saw a curved street surrounded by landscape with trees, buildings and grassland, and were instructed to keep the car centrally in the right lane. The route was a 9 km curved track without oncoming traffic.

#### Manipulation of predictability

**Fig. 1:**
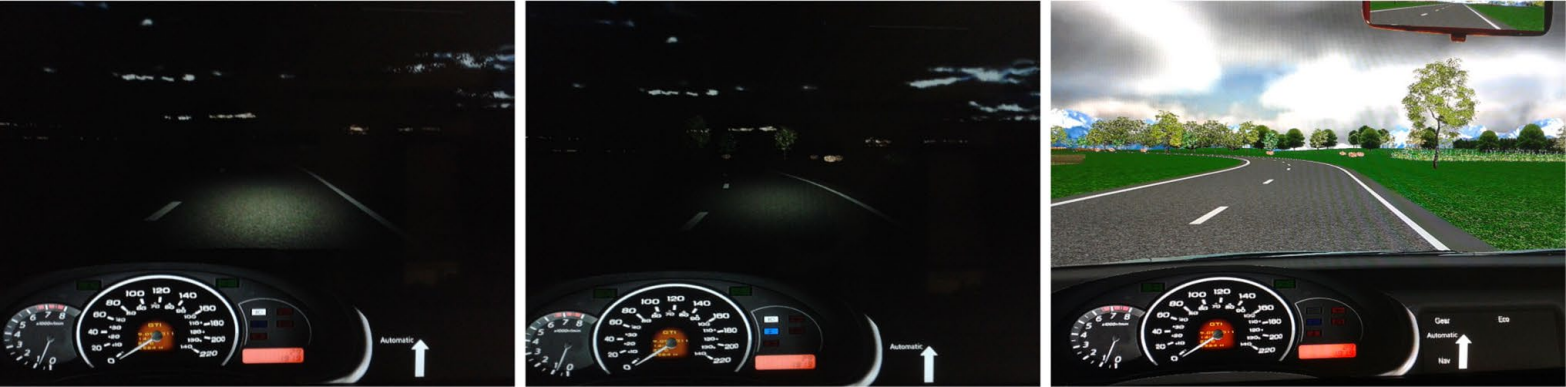
Predictability conditions in the driving task. Participants drove at night with lower beam, at night with high beam or at daylight conditions (from left to right)

Predictability was manipulated via different lights (see Fig. 1). Low predictability was manipulated by a night condition with low-beam headlights, medium predictability was manipulated by a night condition with high-beam headlights, and high predictability was manipulated by a daylight condition.

#### Audiomotor task

Participants had to discriminate between two sounds of 75 ms duration each, a high-pitched target sound (1,086 Hz) and a low-pitched distractor sound (217 Hz). In total, 9–14 target sounds occurred approximately every 138 m along the route, randomly interspersed by distractor sounds. Participants were instructed to disregard distractor sounds, but respond as quickly as possible to target sounds by releasing the gas pedal and pressing the brake pedal until speed decreased to 80 km/h. In single tasks, where participants only had to react to auditory signals, the simulator operated via autopilot and the speed automatically returned to 100 km/h when participants released the brake pedal. For dual tasks, participants had to accelerate back to 100 km/h themselves after braking.

#### Procedure

During the familiarization phase, participants drove a 3-min single-task test drive in daylight condition. They also familiarized themselves with the sounds for 30 s.

The experimental phase of the driving paradigm took about 55 min. The 9-km route took about 7 min and participants performed it seven times in randomized order: three ST driving routes (1 × ST low beam, 1 × ST high beam, 1 × ST daylight); one ST audiomotor route while driving in autopilot mode and three DT routes (1 × DT low beam, 1 × DT high beam, 1 × DT daylight). To signalize the end of each route, a stop sign occurred centrally on screen. Participants were instructed to follow the street, stay centrally in the right lane, to react to target sounds as fast as possible, and to put equal emphasis on both tasks for dual tasks.

#### Data analysis

Driving performance was measured by the standard deviation of the lateral position of the car (SDLP, see Verster and Roth 2011), i.e. from the centre of the right lane in meters. Thus, SDLP = 0 m would indicate that the car keeps a constant distance to the median and to the curb, while high SDLP scores would indicate that the car swerves wildly between the median and the curb. We did not analyse SDLP for the whole track, but for pre-defined intervals which bore relation to target sounds. For every participant, four intervals were analysed: the interval 40 m before, which started 40 m before a target sound occurred and ended at the moment of sound onset. The interval 40 m after ranged from 0 to 40.99 m after sound onset, the interval 80 m after ranged from 41 to 80.99 m after sound onset, and the interval 120 m after ranged 81–120 m after sound onset. The SDLP presented are thus average SD across one interval per condition. Audiomotor performance was assessed as reaction time (RT), defined as the time between sound onset and brake onset.

Subjects were identified as outliers when SDLP in one or more intervals exceeded ± 3.29 SD. This criterion was based on Tabachnik and Fidell (2007) excluding the 0.1% most extreme cases of the data set. These outliers were likely indicators of bad data, resulting from misunderstood instructions, technical problems etc. SDLP was submitted to a 3 × 4 × 2 repeated measures ANOVA with the factors Predictability (three levels: low beam vs. high beam vs. daylight) and interval (40 m before vs. 40 m after vs. 80 m after vs. 120 m after) and task type (ST vs. DT). RTs were analysed by separate *t* tests comparing ST vs. DT_low beam_, ST vs. DT_high beam_, ST vs. DT_daylight_ and a one-way ANOVA comparing the three predictability conditions. Dual-task costs were calculated by the formula $\mathrm{D}\mathrm{T}\mathrm{c}\mathrm{o}\mathrm{s}\mathrm{t}=\frac{({\mathrm{R}\mathrm{M}\mathrm{S}\mathrm{E}}_{\mathrm{S}\mathrm{T}}-{\mathrm{R}\mathrm{M}\mathrm{S}\mathrm{E}}_{\mathrm{D}\mathrm{T}})}{{\mathrm{R}\mathrm{M}\mathrm{S}\mathrm{E}}_{\mathrm{S}\mathrm{T}}} \times 100$ and will be displayed in percentage (Bock 2008).

### Tracking paradigm

#### Setup

Participants were seated in front of a 24″ computer screen (144 Hz, 1920 × 1080 pixel resolution) with a viewing distance of 60 cm. The tracking software ran on a Windows 10, 64-bit system with a GTX750 graphics card. The test room was dimly lit to increase colour intensity and contrast of the monitor. A joystick was positioned in front of the participant on the desk (16-bit Thrustmaster T16000M FCS), and a double pedal was fixed on the floor centrally under the desk (double foot switch Scythe USB 2FS-2). Participants wore headphones (Sennheiser HD 65TV). The experimenter sat in a separated area of the test room to supervise compliance with the task.

#### Visuomotor tracking task

Participants performed a two-dimensional pursuit tracking task with a joystick (adapted from Wulf and Schmidt 1997). They controlled a white cursor cross to track a red target square, which was moving from left to right on a sinusoidal path with constant path speed of 10.5 cm/s. To prevent participants from moving the cursor straight to the right edge of the screen and cut trials short, the cursor cross moved with the same speed as the target on the *x* axis, and so participants could control the white cross by moving the joystick forward and backward only (with the self-reported dominant hand).

Each tracking path consisted of three different random segments from a total set of 41 segments. Each followed the formula (Künzell et al. 2016):

$${b}_{0}+ \sum_{i=1}^{6}{a}_{i} \mathrm{s}\mathrm{i}\mathrm{n}\left(i\times x\right)+{b}_{i} \mathrm{c}\mathrm{o}\mathrm{s}\left(i\times x\right),$$

where *a*_*i*_ and *b*_*i*_ are randomly generated numbers (range of − 5 to 5) and *x* is a real number (range of 0–2*π*). Trial lengths varied from 25.6 to 27.9 s depending on the curve’s amplitude (cf. 27 s used in Raab et al. 2013), which approximate 269–293 cm.

#### Manipulation of predictability

**Fig. 2:**
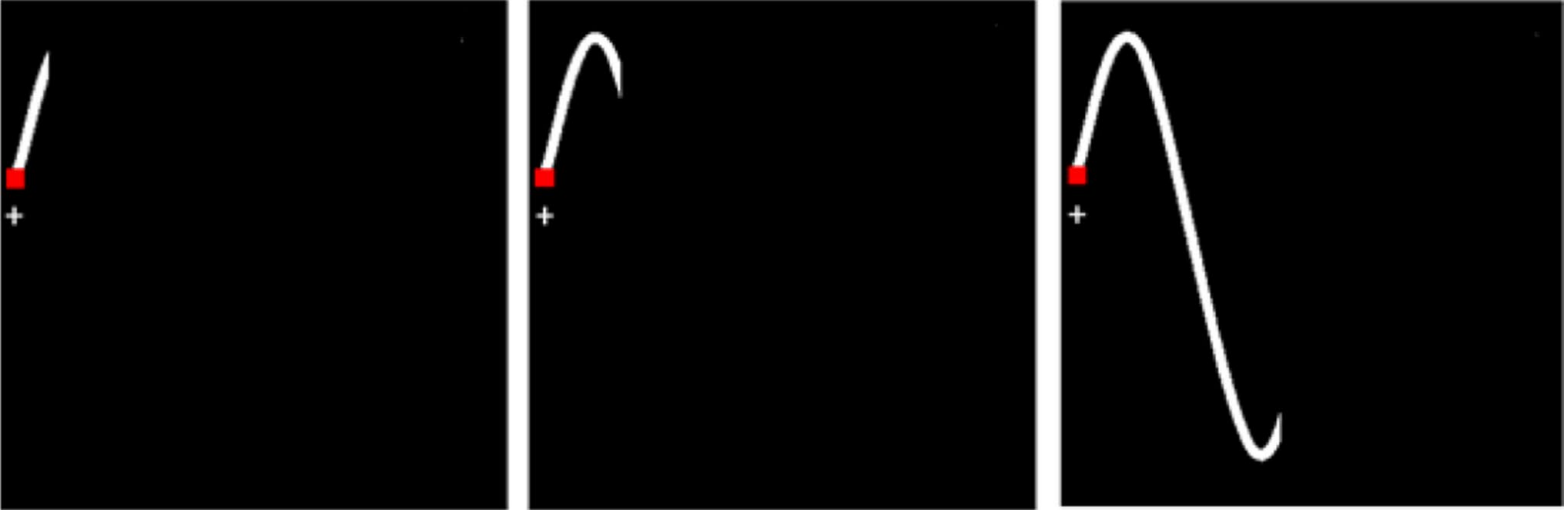
Predictability in the pursuit tracking task. Participants saw a white line of either 200 ms, 400 ms or 800 ms ahead of the target (left, middle, right, respectively)

Predictability was manipulated by visualizing parts of the tracking path ahead of the target. Participants saw a white line ahead of the target which varied between 200 ms (to account for visuomotor delay; e.g., Van Rullen and Thorpe 2001), 400 ms, and 800 ms (see Fig. 2), representing low, medium and high predictability respectively.

#### Audiomotor task

The task was the same as in the driving paradigm. On each path, the minimum gap between two sounds was 1001 ms (see e.g. Bherer et al. 2005), while the first tone appeared no earlier than 500 ms after the start and no later than 500 ms before the end of the tracking path. The number of and distance between sounds were matched to the driving task (every 5–7 s) to ensure comparable levels of difficulty. Valid responses were defined as given within 800 ms after target sound onset.

The audiomotor task in dual-task tracking, however, differed from the driving paradigm in two regards: giving gas was not required, so participants were instructed to only place their right foot on the right pedal and move it to the left to press down the left pedal as fast as possible after hearing the target sound. Second, the tracking cursor did not slow down when participants pressed the pedal, while the driving simulation slowed as a consequence of the braking action.

#### Procedure

To introduce participants to the tasks, they first performed a familiarization phase of two ST tracking trials, two ST audio trials and two DT trials, all of them with no predictability. Participants were asked to be as precise as possible when tracking the target square, to be as fast as possible when reacting to target sounds and to put equal emphasis on both tasks in DT conditions. A feedback window occurred at every five trials providing participants with information about their tracking performance and reaction times (McDowd 1986).

The experimental phase of the tracking part took about 35 min, and as in the driving paradigm participants performed seven blocks with different tracking paths: 30 ST tracking (10 × ST tracking 200 ms, 10 × ST tracking 400 ms, 10 × ST tracking 800 ms); 10 ST audiomotor, 30 DT tracking (10 × DT tracking 200 ms, 10 × DT tracking 400 ms, 10 × DT tracking 800 ms).

#### Data analysis

Tracking performance was measured by the average root mean square error (RMSE; Wulf and Schmidt 1997) and participants’ velocities. Velocities were participants’ speed changes on the vertical axis, and were examined in intervals around the occurrence of target sounds to analyse potential changes in tracking when using the pedal. Like in driving, we created four velocity intervals, translating the relation between the 9-km driving route vs. 280-cm tracking path, and meter vs. milliseconds: 200 ms before sound onset until the moment of sound onset; 200 ms after, which was 75–200 ms after sound onset (given audiomotor delay of 75 ms; Vu and Proctor 2002); 400 ms after, which was 200–400 ms after sound onset; and 600 ms after, which was from 400–600 ms after sound onset. Audiomotor performance was measured by RT, which equivalently to braking responses was defined as the time between sound onset and pedal press onset.

Prior to the analyses, RMSE, velocities and RT were checked for outliers (+ 2 or more standard deviations for each factor). Single participants were excluded from the respective analysis part when four or more trials of a participant within a condition (= 40% as participants completed ten trials per condition) were considered as outliers. Therefore, RMSE analysis consisted of data from all 27 participants, velocities from 24 participants and RT from 26 participants. We will use subscripts to denote conditions, e.g., we will use DT_200_ for dual-task conditions with 200 ms predictability.

RMSE was subject to a two-way repeated measures ANOVA with the factors predictability (200 ms vs. 400 ms vs. 800 ms) and task type (ST vs. DT). Velocities were subject to a 3 × 4 repeated measures ANOVA with the factors predictability (three levels: 200 ms vs. 400 ms vs. 800 ms) and interval (200 ms before vs. 200 ms after vs. 400 ms after vs. 600 ms after). RT was analysed via single *t* tests comparing ST_audio_ vs. DT_200_, ST_audio_ vs. DT_400_, ST_audio_ vs. DT_800_.

## Results

In this study, we aimed at examining the influence of predictability on dual- and single-task driving vs. tracking. As expected, we found that dual-task performance in driving and tracking was worse compared to single-task performance, but predictability mitigates this effect. While in driving, highest predictability was most beneficial and tracking performance was optimal with medium predictability. We further show that patterns of interference between driving and audiomotor task, and tracking and the audiomotor task, was comparable when examining changes of behaviour in the interval where a secondary response was given.

### Driving paradigm

#### SDLP across the whole route (including target and distractor sounds)

**Fig. 3:**
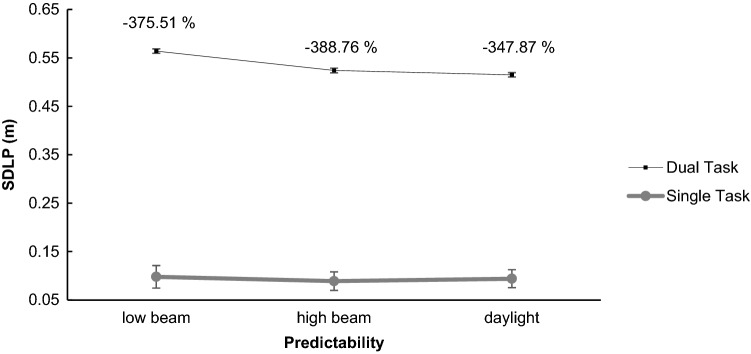
Standard deviation of the lateral deviation in relation to target sounds for single- and dual-task driving. Errors bars show the standard error between participants. DT costs are displayed in percentage

There was a main effect of task type, *F*(1, 26) = 461.01, *p* < 0.001, *η*^2^ = 0.947, because performance was better in single-task driving (> 300% on average). There was also a main effect of predictability, *F*(2, 52) = 8.13, *p* < 0.001, *η*^2^ = 0.238, because across the whole route deviation, the middle of the right lane was lowest for daylight. Post hoc polynomial contrasts show that the relationship between SDLP and predictability was best described by a linear function, *t*(26) = 3.765*, p* < 0.001 (quadratic: *t*(26) = 1.444, *p* = 0.155). There was also a significant task type × predictability interaction, *F*(2, 52) = 4.51, *p* = 0.016, η^2^ = 0.148, showing that the positive impact of increasing predictability was more pronounced in dual-task driving (see Fig. 3).

#### SDLP in four intervals in relation to target sound occurrence

**Fig. 4:**
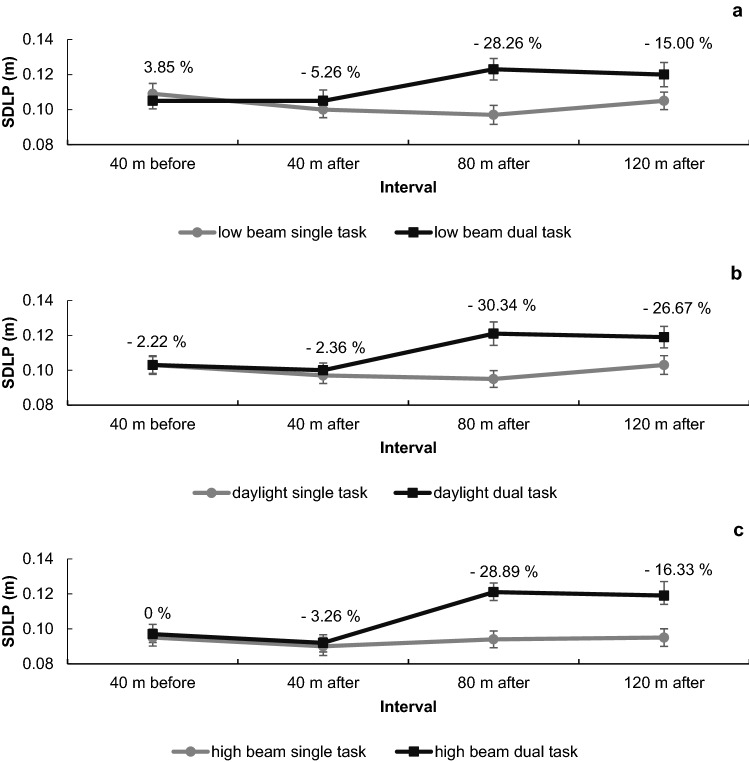
SDLP in relation to target sounds. Black lines depict driving performance in dual tasks; grey lines in single tasks. **a** Driving performance with low beam; **b** driving performance with high beam; **c** driving performance with daylight. Error bars show the standard error between participants. Dual-task costs, which was the difference between single- and dual-task performances, are displayed in percentage

There was a significant main effect of predictability, *F*(2, 52) = 4.60, *p* = 0.014, *η*^2^ = 0.150, because participants deviated least from the middle of the right lane for daylight, and there was a significant main effect of task type, *F*(1, 26) = 28.47, *p* < 0.001, *η*^2^ = 0.523, because lateral deviation was higher in dual tasks. There was also a significant main effect of interval *F*(3, 78) = 23.42, *p* < 0.001, *η*^2^ = 0.474, because participants had highest deviations in the third and fourth interval after target sound onset (see Fig. 3). Post hoc comparisons show that the across predictability conditions, the second and third interval have the largest difference; however, the third and fourth are not different from each other (Table 1). As expected, there was a significant task type × interval interaction, *F*(3, 78) = 21.42, *p* < 0.001, *η*^2^ = 0.452, showing that the deviation from second to third and fourth interval was more pronounced for dual tasks. There was neither a task type × predictability interaction, *F*(2, 52) < 1, *p* = 0.658, *η*^2^ = 0.016, nor an interval × predictability interaction, *F*(6, 156) = 1.53, *p* = 0.171, *η*^2^ = 0.056, nor a significant three-way interaction, task type × interval × predictability, *F*(6, 156) < 1, *p* = 0.822, *η*^2^ = 0.018 (Fig. 4).

**Table 1:** Post hoc comparisons for interval across the three predictability conditions in driving

95% CI of mean difference							
	Mean difference	Lower	Upper	SE	*t*	Cohen's *d*	*p*_bonf_
120 m after							
40 m after	0.013	0.007	0.020	0.002	5.767	1.110	< .001
40 m before	0.008	0.003	0.013	0.002	4.554	0.876	< .001
80 m after	0.002	− 0.003	0.007	0.002	0.976	0.188	1.000
40 m after							
40 m before	− 0.005	− 0.009	− 0.001	0.001	− 3.760	− 0.724	0.005
80 m after	− 0.011	− 0.016	− 0.007	0.002	− 6.731	− 1.295	< .001
40 m before							
80 m after	− 0.006	− 0.011	− 0.002	0.002	− 3.976	− 0.765	0.003

Cohen's *d* does not correct for multiple comparisons. Bonferroni-adjusted confidence intervals

#### RT

**Fig. 5:**
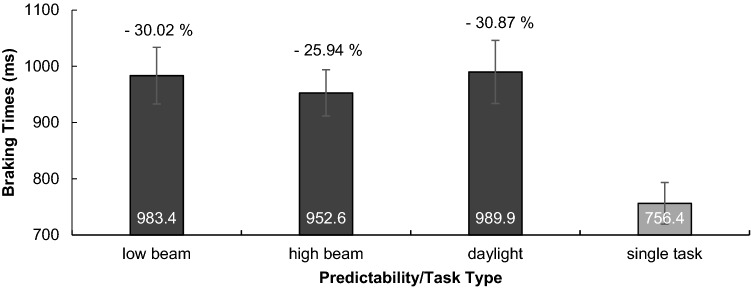
Reaction times in driving (braking times) for the different predictability conditions and for the autopilot single-task in milliseconds. DT costs are displayed in percentage

Time between sound onset and brake onset. There was no significant main effect of predictability on braking reaction times, *F*(2, 52) < 1, *p* = 0.509, *η*^2^ = 0.026 (see Fig. 5).

### Tracking paradigm

#### RMSE across the whole tracking path

**Fig. 6:**
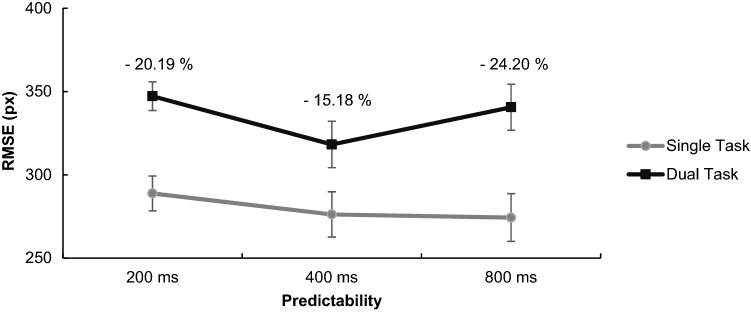
Tracking performance (RMSE) in the different predictability conditions. Error bars show the standard error between participants. DT costs are displayed in percentage

There was a significant main effect of task type, *F*(1, 26) = 37.28, *p* < 0.001, *η*^2^ = 0.589, because participants performed worse in dual-task conditions. There was a significant main effect of predictability, *F*(2, 52) = 5.34, *p* = 0.008, *η*^2^ = 0.170, because performance was best in medium predictability (400 ms). Post hoc polynomial contrasts show that the relationship between RMSE and predictability was best described by a quadratic function, *t*(25) = 2.815*, p* = 0.007 (linear: *t*(25) = 1.658, *p* = 0.103). There was also a significant interaction, *F*(2, 52) = 3.46, *p* = 0.039, *η*^2^ = 0.118, showing that medium predictability had a higher impact on dual-task conditions (see Fig. 6).

#### Velocities in four intervals relation to target sound occurrence

**Fig. 7:**
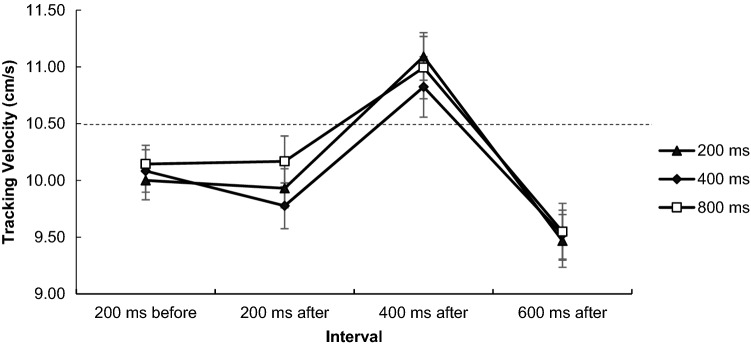
Velocity analyses for tracking. Baseline tracking velocity (200 ms before the occurrence of a target sound) was compared against 200 ms, 400 ms, and 600 ms after the sound onset. The dashed horizontal line represents the constant target velocity (10.5 cm/s). Error bars show the standard error between participants

Velocities are the mean changes in tracking speed in relation to the occurrence of target sounds. There was a main effect of interval, *F*(3, 72) = 29.48, *p* < 0.001, *η*^2^ = 0.551, showing that participants significantly changed tracking speed in the third interval (400 ms after) after target sound onset (see Fig. 7). Post hoc comparisons confirm that the difference between the second and the third interval was significant (see Table 2). There was neither a main effect of predictability, *F*(2, 48) = 1.38 *p* = 0.260, *η*^2^ = 0.055, nor a significant interaction, *F*(6, 144) = 1.26, *p* = 0.281, *η*^2^ = 0.050.

**Table 2:** Post hoc comparisons for interval across the three predictability conditions in tracking

95% CI of mean difference							
	Mean difference	Lower	Upper	SE	*t*	Cohen's *d*	*p*_bonf_
200 ms after							
200 ms before	− 0.117	− 0.549	0.314	0.159	− 0.737	− 0.147	1.000
400 ms after	− 1.012	− 1.443	− 0.580	0.159	− 6.358	− 1.272	< .001
600 ms after	0.446	0.014	0.878	0.159	2.802	0.560	0.039
200 ms before							
400 ms after	− 0.894	− 1.326	− 0.463	0.159	− 5.621	− 1.124	< .001
600 ms after	0.563	0.131	0.995	0.159	3.539	0.708	0.004
400 ms after							
600 ms after	1.457	1.026	1.889	0.159	9.160	1.832	< .001

Cohen's d does not correct for multiple comparisons. Bonferroni-adjusted confidence intervals

#### RT

**Fig. 8:**
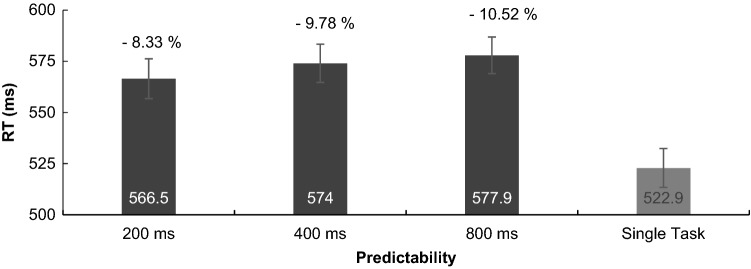
Reaction times in the tracking task for the three predictability conditions and the single-task condition. Error bars show the standard error between participants. DT costs are displayed in percentage

There was no effect of predictability on RT, *F*(2, 52) = 2.76, *p* = 0.072, *η*^2^ = 0.096, because RTs did not differ between the conditions 200, 400 and 800 ms (see Fig. 8).

### Correlations

We did not endorse significant correlations worth reporting between the dependent variables of the two tests (i.e. RMSE vs. SDLP across the whole route; tracking velocity in four intervals vs. SDLP in four intervals). For further information consult the “Appendix”.

## Discussion

In this study, we aimed at examining the influence of predictability on complex dual-task driving and on a basic continuous tracking task. Summing up the results, we showed that predictability can have a positive impact on performance, both in driving and in tracking. In either task, visual information did, however, not impact performance on the auditory task (neither reaction nor braking times differed between predictability conditions), so we did not endorse a beneficial impact of primary-task predictability on secondary-task performance. It was noteworthy that both tracking velocities and SDLP in relation to target sound responses yielded a comparable pattern: participants exhibited major changes in tracking/driving behaviour between the second and the third interval after sound onset (40–80 m after and 200–400 ms after, respectively) in dual-task conditions. Even the size of the response times was different, the third intervals were comparable and corresponded roughly to moments of response selection (conceivably also response execution) and thus, as we will discuss later, the increased deviation/velocity may be an indication of interference between tasks.

In detail, results demonstrated that driving performance, as one would intuitively predict, improved with increasing predictability: it was best in single-task daylight condition and worst in dual-task night low-beam condition. Considering SDLP across the whole route (incl. distractor and target sounds), predictability in the first task positively impacted dual-task driving and highest predictability was most beneficial, which was substantiated by polynomial contrasts. This was in contrast to results obtained for tracking, where medium predictability was most beneficial to performance as predicted by previous tracking studies (Broeker et al. 2020; de Oliveira and Wann 2012). It is not clear why high predictability was most beneficial to driving and medium predictability most beneficial to tracking. We speculate that most participants were unfamiliar with tracking and integrating two-dimensional visual information, yet all of the participants were drivers and, therefore, used to driving in daylight where looking (far) ahead and integrating many information are well practised and most important to security. Besides, research in motor learning has shown that principles derived from the learning of simple tasks cannot be simply transferred to the learning of complex tasks anyways (Hill and Raab 2005; Wulf and Shea 2002). According to that, increasing motor demands are associated with an increased need for visual information and feedback (Gray 2008), so it may not be surprising that also predictability and visual information for visuomotor control in basic tracking vs. complex steering tasks are used differently. In this vein, it was also interesting to observe that braking times were almost twice as high as reaction times in tracking, thus task complexity or higher visual processing might have slowed down braking times in general. We can, however, not rule out that a share of this difference resulted from different resistances of the tracking and braking pedal, or the different instructions and participants’ related associations of “braking as fast as possible” vs. “pressing as fast as possible”. We may also speculate that our instruction to brake down to 80 km/h enticed participants to react more slowly compared to a hazard braking instruction. What we can, however, conclude is that no significant differences between braking/reaction times for predictability conditions, and also no differences in dual-task costs between conditions speak against a beneficial impact of primary-task predictability on secondary-task performance. The unrelatedness of the secondary task may be crucial to this absent transfer effect of predictability. Previous dual-task studies that, for instance, covaried tracking and auditory tasks (e.g. by letting target sounds announce turning points of the tracking path) showed an increased performance in both tasks, possibly because they get a meaningful relation and can be better integrated (de Oliveira et al. 2017; Schmidtke and Heuer 1997).

What both tasks also shared was the relatively high deviation in manual accuracy between the second and third interval after target sound onset, which corresponded to the moment the pedal response was given. This contrasted both previous driving studies where lane maintenance was not affected by secondary tasks (e.g. Özbozdağlı et al. 2018), and classic dual-task studies which typically report secondary task performance to be affected. So, in our study there seems to be a universal effect of motor interference when concurrently performing a visuomotor and audiomotor task that occurs independent from task complexity. The extent of the interference was not impacted by predictability, so more predictability did not mean lower deviation as indicated by non-significant interval × predictability interactions in driving and tracking. Resource sharing accounts (Donk and Sanders 1989; Wickens 2008) would argue that hands and feet draw on the same motor resource and that participants encounter some resource depletion when both tasks have to be responded to, irrespective of the visual information present. Bottleneck accounts would argue that interference occurred when the tracking task and audiomotor task overlap at the central response selection stage. According to the original theory and its applications in psychological refractory period (PRP) paradigms, any peripheral stage (e.g. perception and motoric response) of Task 1 can proceed in parallel to stages of Task 2, but Task 2 response execution is delayed due to competition at the response selection stage, which itself is neither sensory nor effector specific (Fischer and Plessow 2015; Pashler 2000). Levy et al. (2006) and Levy and Pashler (2008) assessed effects on central processing in driving and found PRP effects on braking RTs. They proposed that two concurrently performed tasks race for the bottleneck, with the losing task having higher RTs. They also found that preceding redundant signals coupled to the braking signal can afford faster processing contributing to the likelihood that the braking task would win the race and mitigate the PRP effect. The latter finding would be consistent with our study in the sense that distractor sounds served as redundant signals and enabled faster responses, nonetheless we found interference in driving/tracking (equivalent to Task 1), and not in T2 braking times like Levy et al. According to their theoretical position, we would conclude that the auditory task temporarily occupied the bottleneck and won the race, which has led to temporary performance losses in driving/tracking, even though predictability should have enabled faster processing for the driving/tracking task and prevented this interference. One difference between the studies is that Levy and Pashler (2008) explicitly instructed participants to prioritize driving, so our instruction of equal emphasis might have facilitated the auditory task to “win the race”; explaining the opposite effect. Another difference to their study was that Levy and Pashler manipulated pre-defined stimulus onset asynchronies (SOAs). Given that in both of our paradigms, sounds and inter-stimulus intervals were random, and there were no imperative stimuli coupled to the continuous driving/tracking task, we could not identify SOAs which makes it hard to disentangle perceptual, processing, and execution stages for the primary task. It, therefore, remains speculative whether the performance loss in the third interval was due to the auditory task occupying the response selection stage. We think it should be critically discussed whether continuous tasks that require permanent and (possibly parallel) response selection and execution allow for stage logic and the identification of PRP effects per se. There are, however, so-called motor-bottleneck accounts which argue that a motor requirement of the first task (i.e. response) creates a general motor refractory period that prevents the initiation of the next motor response for task two (Bratzke et al. 2009). The initiation of a pedal press might have thus led to a rapid channel switching, temporarily extending the PRP period of the first task which would explain the interference in tracking. Both explanations being either that the audiomotor task has taken away resources from the continuous task, or that it temporarily occupied a channel, seem possible but remain abstract, metaphoric concepts are currently not testable with at least one task that is not time locked but continuous. Irrespective of the underlying mechanism explaining the interference, it remains important that predictability can overall improve dual-task performance, yet not the extent of the interference per se. An information we have only been able to observe by analysing spatio-temporal variables beyond spatial variables like RMSE.

There were some limitations that need to be discussed. Future studies might consider longer exposure to the driving task. In our design, the tracking paradigm took 35 min, while the driving part was 55 min and we expected this difference to be large enough to account for the need for more extensive practice in the complex task, but results might change for longer driving experience. Also, the decision to use 200, 400 and 800 ms as visual information in tracking was based on previous own research, but the claim that e.g. 400 ms would be comparable to a high beam or daylight remains speculative and other manipulations of the driving scenery should be tested (e.g. different fog densities but same light, all night conditions but different beams, etc.). Numerous previous experiments of our group have shown that 400 ms is optimal for visuomotor performance in tracking tasks (for a discussion see Broeker et al. 2020), but there was no evidence on how much visual advance information in complex scenery in meters or milliseconds would be most beneficial to visuomotor control. Finally, the dropout rate was higher than expected and we tested three participants less than required to fulfil a priori power estimations, so different results might occur for a larger sample.

## Appendix

### Correlations

Correlation between tracking performance (RMSE) and driving performance (mean lateral deviation) in single and dual tasks.

**Table:**

	Single task			Dual task		
	Night low beam	Night high beam	Daylight	Night low beam	Night high beam	Daylight
200 ms	0.07	0.13	0.19	0.36	0.20	0.37
400 ms	0.37	**0.43***	0.37	0.39*	0.20	0.39*
800 ms	0.31	0.39*	0.36	0.29	0.12	0.34

Bold values refer to a significant correlation between two matching categories

****p* < 0.001, ***p* < 0.01, **p* < 0.05

Correlations between tracking velocities and driving performance (mean lateral deviation in relation to target sounds) for dual tasks.

**Table:**

	40 m before	40 m after	80 m after	120 m after
200 ms/night low beam				
200 ms before	− 0.07			
200 ms after		− 0.17		
400 ms after			0.35	
600 ms after				− 0.27
400 ms/night high beam				
200 ms before	0.03			
200 ms after		0.10		
400 ms after			0.25	
600 ms after				− 0.01
800 ms/daylight				
200 ms before	**0.00**			
200 ms after		− 0.08		
400 ms after			0.10	
600 ms after				− 0.33

Bold values refer to a significant correlation between two matching categories

****p* < 0.001, ***p* < 0.01, **p* < 0.05
